# Poster Session II - A217 BUTYRATE’S ROLE IN MODULATING TIGHT JUNCTIONS, GPR41/43 RECEPTORS, AND IMMUNOGLOBULIN RESPONSES IN EXPERIMENTAL COLITIS

**DOI:** 10.1093/jcag/gwaf042.217

**Published:** 2026-02-13

**Authors:** F Ait Zenati, M Sabzevary, J Ghia

**Affiliations:** Immunology, University of Manitoba, Winnipeg, MB, Canada; Immunology, University of Manitoba Max Rady College of Medicine, Winnipeg, MB, Canada; Immunology, University of Manitoba Max Rady College of Medicine, Winnipeg, MB, Canada

## Abstract

**Background:**

Inflammatory bowel disease (IBD) involves impaired mucosal barrier function, cytokines, and dysregulated B-cell responses. Emerging evidence suggests that the gut microbiota and its metabolites, including short-chain fatty acids (SCFA) like butyrate, modulate intestinal inflammation. However, the specific role of butyrate via its epithelial receptors in regulating mucosal permeability and B-cell activity remains unclear. We hypothesize that butyrate influences epithelial barrier integrity and B-cell function during colonic inflammation.

**Aims:**

To determine how butyrate modulates epithelial tight junctions, GPR41/43 receptor activation, and mucosal immune responses, including lumen immunoglobulin release, in experimental colitis.

**Methods:**

C57BL/6 mice (n = 8/group) received 150 mM sodium butyrate in drinking water for 30 days before inducing colitis with 5% dextran sulfate sodium (DSS) for 5 days. Disease activity index (DAI), colon length, and macroscopic scores were recorded to evaluate colitis severity. Immunofluorescence staining was performed on mid-colon segments to assess epithelial Zonula Occludens-1 (ZO-1) and butyrate receptors GPR41 and GPR43 expression. Serum and colon wash samples were analyzed for immunoglobulin isotypes (IgA, IgG1, IgM IgG2b) using ELISA, in addition to colonic and serum TGF-b, and colonic TNF-a and IL-17.

**Results:**

DSS-induced colitis significantly increased DAI and macroscopic damage and reduced colon length compared to controls. Butyrate-treated colitic mice demonstrated a higher DAI on day 5 than colitic mice, without improvement in colon length or macroscopic pathology. Butyrate significantly increased ZO-1 immunofluorescence in both control and colitic mice. In parallel, expression of butyrate receptors GPR41 and GPR43 was elevated in both colitic and non-colitic butyrate-treated mice, with the highest levels observed in colitic group. Systemically, colitis significantly decreased serum IgG2b. In both colitic and non-colitic mice, butyrate treatment did not alter serum immunoglobulin isotypes. In colon washes, colitis significantly increased serum IgA, and butyrate treatment significantly decreased IgG1 a level in colitic mice. Serum and colonic levels of TGF-b were not modified in colitic and non-colitic mice, regardless of the treatment. However, in colitis, butyrate treatment decreased significantly colonic TNF-a and IL-17.

**Conclusions:**

Together, these findings suggest that butyrate enhances epithelial barrier function by upregulating ZO-1, but has limited effects on systemic immunoglobulin responses, while modulating local IgG1 and pro-inflammatory cytokines in colitis. Our results support a model in which butyrate provides protective effects on mucosal immunity. These insights could inform the development of microbiota and SCFA-based therapeutic strategies for IBD.

**Funding Agencies:**

NSERC

